# A multi-centre, randomised controlled trial of the effectiveness of PDSAFE to prevent falls among people with Parkinson’s: study protocol

**DOI:** 10.1186/s12883-015-0332-2

**Published:** 2015-05-15

**Authors:** Victoria A Goodwin, Ruth Pickering, Claire Ballinger, Helen Roberts, Emma McIntosh, Sarah Lamb, Alice Nieuwboer, Lynn Rochester, Ann Ashburn, on behalf of the PDSAFE Protocol Development Group

**Affiliations:** University of Exeter, Exeter, UK; University of Southampton, Southampton, UK; University of Glasgow, Glasgow, UK; University of Oxford, Oxford, UK; University of Leuven, Leuven, Belgium; Newcastle University, Newcastle, UK

**Keywords:** Parkinson’s falls, Randomised, Physiotherapy, Exercise, Strategies

## Abstract

**Background:**

Falls amongst people with Parkinson’s (PwP) result in significant disability and reduced quality of life. There is emerging evidence that exercise-based and physiotherapeutic interventions are of benefit for improving fall risk factors, such as balance. However, the benefit, in terms of preventing falls, is mixed. The development of effective interventions has been identified as the highest research priority for this population.

The aim of this trial is to establish the effectiveness and cost-effectiveness of a novel, home-based physiotherapy programme, compared with usual care, on falls amongst PwP.

**Methods/Design:**

A UK multi-centre, community-based, single blind, randomised controlled trial with twelve month follow-up, and nested economic evaluation and qualitative studies will be undertaken. Six hundred PwP who live in their own home, have had one or more falls in the previous year and an MMSE score of ≥24 will be recruited. Those living in care homes and those needing assistance from another person to walk indoors will not be eligible.

The intervention is a physiotherapist delivered, individually tailored and progressive, home-based programme (PDSAFE) comprising task orientated movement strategy training, functional lower limb strengthening and balance training, of six months duration. Unsupervised daily home exercises and strategies will be practised and supported using technology. Control participants will receive usual care.

Data collection will include falls, cognitive state, balance and mobility, fear of falling, freezing of gait, mood, quality of life, carer quality of life and resource use. Data will be collected at baseline, three, six and twelve months. Longitudinal semi-structured interviews will be undertaken with forty participants to explore the expectations and experiences of participants.

The primary outcome is risk of repeat falling at six months post-randomisation.

**Discussion:**

The aims of this trial are to establish the effectiveness and cost-effectiveness of a novel, home-delivered physiotherapy intervention (PDSAFE) compared with usual care on risk of falling for PwP who have a history of falling. PDSAFE is a novel intervention that builds upon the existing literature and targeting known risk factors, being the first study that uses a novel delivery modus (technology) in conjunction with traditional physiotherapeutic approaches.

**Trial registration:**

Current Controlled Trials ISRCTN48152791

## Background

In the UK, there are estimated to be around 127,000 people with Parkinson’s (PwP) [1]. The lifetime prevalence of Parkinson’s in the UK is two per thousand with an annual incidence of nineteen per hundred thousand population [2]. PwP are twice as likely to experience falls when compared with the community-dwelling, older population due to both physical and cognitive impairments [3]. Falls and fall-related injuries have not been identified separately within cost of illness studies in Parkinson’s [4] but contribute to morbidity, hospital admissions and reduced quality of life in this population [5,6]. A national survey among the national Parkinson’s Foundation Centres in the USA listed falls and fractures as one of the top reasons for hospitalisation in this population [7]. Similarly, an Australian study reviewed 645 admissions in which Parkinson’s was a secondary diagnosis and identified falls and related fractures as the leading reason for hospitalisation accounting for 13% of the total cases [8]. A recent survey of a thousand PwP, carers, and health and social care professionals reported the development of interventions to reduce falls and improve balance as the highest research priority [9].

A UK survey involving over 13,000 PwP indicated that only 54% had ever seen a physiotherapist as part of their management [10]. The prevention of a cycle of inactivity and injurious falls is a global priority [11]. Despite a wealth of evidence of effective interventions, in particular exercise, for reducing falls in the general older population [12,13], the evidence to date for the prevention of falls for PwP is mixed [14]. People with reduced balance control and falls respond poorly to medication, the mainstay of Parkinson’s management [15]. In a randomised controlled trial of home-based physiotherapy with 142 participants, Ashburn et al. [16] demonstrated a non-significant difference in fall risk at six months of −5% (95 % CI −20 %, 10 %). Goodwin and colleagues [17] compared a physiotherapist-led group strength and balance programme plus additional home-based exercises with usual care and reported a fall incident rate ratio (IRR) of 0.74 (95 % CI 0.41, 1.33) at 20 weeks. A recently published trial from Australia evaluating an intervention that comprised progressive strength and balance training and cueing strategies for six months also reported a non-significant reduction in falls, however subgroup analysis indicated the programme was effective for those with lower disease severity [18]. An RCT of Tai Chi for those with mild Parkinson’s was effective at reducing falls as a secondary outcome [19]. Despite the limited evidence for reducing falls, there is evidence of the benefit of exercise on fall risk factors, such as impaired balance [20] and of movement strategies to reduce freezing [21]. There is also a high probability of exercise interventions being cost-effective for PwP [22].

PwP have different risk factors for falling than the general older population, including freezing of gait, cognitive impairment and poor mobility [23]. A recent paper [24] reported that ambulatory activity and the context in which people fall contributes to the heterogeneous nature of falls in PwP and that “one size fits all” interventions are unlikely to be beneficial. Interventions therefore need to be multimodal and individually tailored in order to be effective.

The aim of this trial is to establish the effectiveness and cost-effectiveness of a novel, home-based physiotherapy programme called PDSAFE, compared with usual care, on falls amongst PwP. A nested longitudinal qualitative study additionally seeks to explore the views, expectations and experiences of trial participants, in order to further explain findings of the trial.

## Methods/Design

### Trial design and setting

The trial is a UK multi-centre, community-based, single blind, randomised controlled trial with twelve month follow-up, with nested economic evaluation and qualitative studies. The trial will be conducted at four sites: Southampton, Portsmouth, Bournemouth /Poole and Exeter. The primary outcome is risk of repeat falling over the 6 month period. Ethical approval has been given by the National Research Ethics Service - South Central – Hampshire B (Reference 14/SC/0039). Trial registration reference is ISRCTN48152791.

### Participants

Participants will be eligible for the trial if they: (a) have a confirmed consultant diagnosis of Parkinson’s; (b) live at home; (c) have had one or more falls in the previous year; (d) score ≥24 of the Mini-Mental State Examination (MMSE) [25]; (e) are able to understand and follow instructions; (f) are able to undertake an exercise programme; (g) are able to give informed consent. Exclusion criteria are: (a) living in a care home; (b) need assistance from another person to walk indoors; or (c) wheelchair bound or bedridden unless aided as defined by Hoehn and Yahr Stage 5 [26].

### Sample size

**Figure 1:**
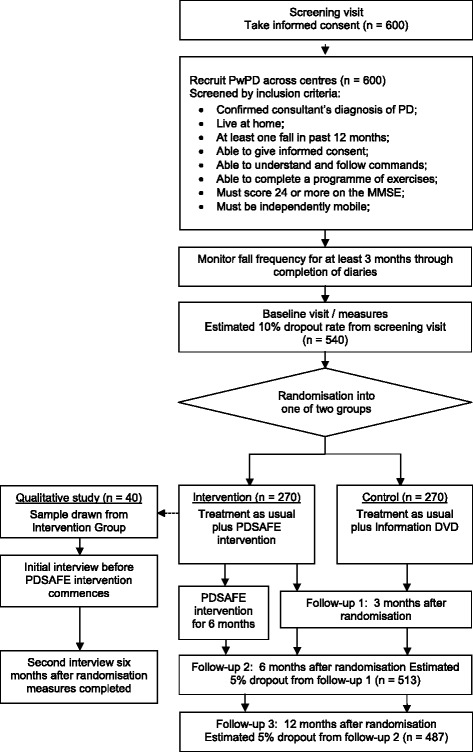
Trial flow diagram

The primary outcome is the risk of repeat falling between 0–6 months. In a previous trial of PwP with two or more falls in the previous year [16], the risk of repeat falling at six months was 68 % (42/63) for controls versus 56 % (35/63) in the exercise group. However, the population in the proposed trial will include those with one or more previous falls and we therefore anticipate the risk of repeat falling to be lower. Assuming a control group risk of repeat falling between 0–6 months to be 63 %, and 50 % in the intervention group, a sample size of 228 participants per group (n = 456 total) with data for analysis is indicated. Allowing for 10% drop out between agreeing to the 3 months pre-randomisation fall data collection and randomisation, and 5 % of loss to follow-up at 6 months amongst those randomised, a total of 534 participants need to be recruited. We aim to recruit 600 participants. Figure 1 provides a flow diagram showing anticipated numbers at recruitment and follow-up.

### Recruitment

Potential participants will be identified from clinical registers of PD specialists at each site, research registers of those who have previously indicated interest in taking part in future research, and from Parkinson’s UK local groups, supported by the National Institute for Health Research (NIHR) Clinical Research Network (CRN). Those identified from clinical registers will be invited to participate, in writing, by their specialist, and those expressing an interest will be asked for permission to pass their contact details onto the research team in order that further information can be shared, and the opportunity to participate offered. All potential participants will be provided with a Participant Information Sheet, a reply slip and pre-paid envelope. On receipt, those expressing interest will be contacted by telephone by the research team to confirm eligibility and offered a home visit (allowing at least 24 hours for consideration before proceeding) to gain written informed consent and complete screening measures. Once consent is gained, demographic data, medical history, MMSE, Montreal Cognitive Assessment (MoCA) [27], Hoehn & Yahr score and number of falls in previous year will be collected and participants will be given diaries to complete for three months to prospectively record any fall events. Permission at this time will also be sought to pass details of those randomised to the intervention arm to a qualitative researcher in order that contact can be made to discuss involvement within the nested qualitative study (see below). Where appropriate, carers will also be invited to participate in the study and will be provided with a carer information sheet. Recruitment commenced in July 2014.

### Randomisation

Following baseline assessment, individuals will be randomised in a 1:1 ratio to either intervention or control arms, stratified by site, using random block size of 2, 4, 6 or 8 to ensure balance within each site, whilst maintaining allocation concealment. The aim will be to undertake the randomisation between thirteen and sixteen weeks post baseline assessment after the completion of three months of pre-randomisation fall diaries. The process will be coordinated by the UKCRC registered Oxford Clinical Trials Unit. To ensure concealment, study allocation will be undertaken using a password protected, web-based portal with allocation only generated after registration of each eligible participants. Randomisation outcome will be e-mailed to the trial co-ordinating centre and the details of allocation will be forwarded to the physiotherapy team who will advise on the timescale for their first visit which should be within two weeks (maximum of four weeks).

### Interventions

All trial participants will continue to receive usual care as deemed by their care providers, including medical and nurse specialist assessment and interventions, such as medication management, as well as usual activities, such as exercise or social groups. They will be encouraged to avoid changing their activity unless specifically advised by a healthcare professional, as well as keep a record of their usual care and activity and any exercise classes outside of the trial for the duration of the study.

The central component of the PDSAFE intervention is a physiotherapist delivered, individually tailored, progressive home-based exercise programme to target modifiable risk factors for falls, including freezing, balance and mobility impairment and physical activity. The programme was informed by the following work – The FaME programme [28], the EXSart Trial [16], the Otago Exercise Programme [29], the PD-WEBB programme [18], the GETuP trial [17], David et al. [30], Mayer et al. [31] and the American College of Sports Medicine [32]. The programme will be delivered by physiotherapists who will undertake an assessment to identify impairments and activities that will be addressed within the intervention programme. The programme will comprise a warm up session and three main active elements:

- Warm up and major muscle group stretches to prepare the body for exercise and enable the body to move more freely when exercising.
- Task-orientated movement strategy training to improve freezing of gait and performance of complex tasks specific to fall-related activities and circumstances within the home environment. A context and task specific evaluation of falls risk will be undertaken to identify specific problematic areas and strategies identified to address the problems. Videos will be recorded by the therapist using a tablet of the participant engaged in activities with and without the use of strategies. Training will include activities such as turning, and single and dual tasks of complex functional activities. Tailored video vignettes of strategies will be given to participants on a DVD to remind and reinforce between face-to-face sessions. This type of technology has been shown to be of benefit for delivering fall prevention exercises [33]. Participants will be asked to practice the strategies daily and to integrate these into daily functional tasks where possible.
- Functional lower limb strengthening exercises will be progressive using bodyweight or a weighted vest, maintaining intensity at a moderate-hard level as assessed by the Borg Scale (Borg 1987).
- Dynamic balance exercises will be progressed through more complex activities and varied starting positions, postures and cognitive challenges maintaining difficulty at a moderate to hard level.

An individualised booklet of exercises will be provided, selected from a menu following assessment. If an individual struggles in achieving a good technique the physiotherapist will video record them doing the exercises correctly using a tablet device. This recording will be shared with the participant to provide them with an accurate visual reference when practising. Participants will be encouraged to perform all exercise components daily. However, to avoid the possibility of overload, a daily safety questionnaire is completed, that advises participants which components to complete and which to leave, due to the risk of fatigue or other safety issues.

The intervention will take place over a six-month period and will consist of up to twelve, one hour face-to-face sessions with the physiotherapist. These face-to-face sessions will be delivered more intensely to start but less frequently over time to maximise motivation and promote independence. The exact delivery of these, however, will be negotiated between the physiotherapist and participant. This model of delivery is unique in exercise studies for PwP. An example delivery model could be:

- Twice a week for weeks 1–2
- Once a week on week 3, 4, and 5
- Once a month at 2, 3, 4, 5 and 6 months.

Participants in the control group will continue to receive usual care and will be advised to continue any activities in which they usually participate, but not to commence new activities, if possible. In addition, they will be visited once by a physiotherapist within four weeks of completing pre-randomisation fall diaries and provided with a DVD about Parkinson’s. After the twelve month follow-up assessment has been completed, they will receive written and verbal guidance on physical activity and strategies by the physiotherapist.

### Physiotherapist training

Physiotherapists will deliver the intervention in participants’ homes across the four study centres. In order to minimise inter-therapist variation and enhance fidelity, a detailed training programme has been developed that includes clinical reasoning for prescribing the exercises and strategies and practical delivery of the programme. This is delivered in a two-day face-to-face session. Once trained and delivering the intervention there will be regular team meetings including masterclass sessions, case studies, and weekly telephone support from the lead physiotherapist.

### Data collection

#### Pre-randomisation fall data

Participants will be asked to prospectively record falls using a monthly diary for the three months between recruitment and randomisation. This process will enable participants to become familiar with the procedure for recording falls and assist the research team with identifying those participants who may need additional support to complete the diaries. In addition, the data will be used in the main trial analysis as a covariate.

### Baseline assessment

On completion of three months of pre-randomisation fall data, an assessor will visit the participants (and carer) at home to complete baseline data collection (Table 1) comprising functional activities, Parkinson’s severity, freezing, quality of life, depression, fear of falling and physical activity. Information about current medication, social status and rehabilitation input will also be collected. This assessment will be undertaken prior to randomisation and the assessor will be blinded to subsequent allocation.

**Table 1:** Data Collection

	Method	Screening (3 months pre-baseline)	Baseline	3 months	6 months	12 months
MMSE [25]	Researcher assessed	x				
Hoehn and Yahr Scale [26]		x				
MoCA [27]		x				
Mini BesTest [53]			x	x	x	x
Timed Chair Stand [54]			x	x	x	x
MDS-Unified Parkinson’s Disease Rating Scale (UPDRS) - motor scale [55]			x	x	x	x
Falls Efficacy Scale International [56]	Self-report (face-to-face)		x	x	x	x
EuroQoL (EQ-5D) [37]			x	x	x	x
New Freezing of gait questionnaire [57]			x	x	x	x
Parkinson’s Disease Questionnaire 39 [58]			x	x	x	x
Physical Activity Scale for the Elderly [59]			x	x	x	x
Geriatrics Depression Scale [60]			x	x	x	x
Carer Experience Scale (CES) [38]			x	x	x	x
Carer Strain Index (CSI) [39]			x	x	x	x
Health and Social Care resource use			x	x	x	x
Falls, near falls, fractures		x				
Adverse events		x				

### Follow-up assessment

Follow up data (Table 1) will be collected at three, six and twelve months post-randomisation during a face-to-face visit to the participants’ home by an assessor blinded to group allocation. Serious adverse events (SAE) have been defined as hospitalisation as a result of a fall directly linked to the treatment with a therapist or whilst doing it on their own. Previous experience within the team suggests that participants may inadvertently unblind assessors. We aim to minimise this through explicit reminders prior to assessment visits. We will also ask assessors to record their estimate of which study arm they believe participants have been allocated to, and their confidence in that belief at the end of the trial. This will enable us to examine the extent of unblinding. Assessments will last approximately 90 minutes and, where possible, all assessments for an individual will be arranged for a similar time of day at mid-medication cycle when movement is optimal.

Fall data will be collected using monthly, prospective, self-report diaries for twelve months following randomisation and will include falls, near falls and injuries [34]. In addition to the diary, participants will be provided forms to completed details of any falls such as location and subsequent treatment [35]. The diaries will be delivered by the assessor at assessment visits and returned each month in a pre-paid envelope and with telephone reminders when not received within three weeks.

The implementation of the intervention protocol in terms of quality and dose will be monitored [36]. Joint patient sessions will be undertaken with the lead physiotherapist to assess intervention fidelity. Participant adherence to the unsupervised programme will be established by them completing a daily tick sheet of the exercises they have completed.

For the economic evaluation, resource use (health and social care) and carer data will be collected using a tick box resource sheet aided by a resource use diary. Additional resource use will be collected within the fall dairies. Information on hospital admissions, length of stay, ambulance call outs, A&E attendances, GP and specialist appointments, physiotherapy, occupational therapy and admission to a care home will be identified and measured. Costs of implementing PDSAFE include training, time to deliver the intervention (face-to-face and telephone), travel, equipment and DVD production will also be identified and measured. Participant quality of life (QOL) data will be collected using the EuroQOL EQ-5D instrument [37] during assessment visits to enable the measurement of Quality Adjusted Life Years (QALYs). To capture any potential spillover QOL impacts carer data will collected using the Carer Experience Scale (CES) [38] and the Caregiver Strain Index (CSI) [39].

### Statistical analysis

The main analysis will be based on intention to treat in that participants will be analysed according to the group to which they were allocated irrespective of the extent of intervention received. The primary outcome, risk of repeat falling over the 6 month period after randomisation, will be compared between intervention and control groups using a logistic regression model including falling during the pre-randomisation monitoring period, Hoehn and Yahr score, and centre as covariates, with the definition of the falling covariate decided at blind review of the data prior to analysis.

Repeat falling during 6–12 months and other binary secondary outcomes will be examined using logistic regression models similar to that for the primary outcome. The rates of falling over 0–6 months and over 6–12 months will be examined in negative binomial models including baseline rate of falling over the 3 month pre-randomisation falls collection period, Hoehn and Yahr score, and centre as covariates, fitted using either the *nbreg* or *xtpoisson* regression commands in Stata (Statacorp, TX, USA). In the negative binomial model, the effect of the intervention is summarised as a falls incidence rate ratio (IRR) (intervention/control) with ratios below 1.00 indicative of lower rates in the intervention group. All participants in the main trial will have been asked to complete the baseline three months falls collection, and the length of follow-up time over which falls events are collected between 0–6 months and 6–12 months post randomisation will be included as exposure times in the regression. Rates of falling in each of the three month periods between the pre-randomisation period and 12 months in the intervention and control groups will be displayed graphically. Other secondary outcomes will be examined in mixed normal models for repeated measurements at 3, 6 and 12 months controlling for centre, Hoehn and Yahr score, and baseline value, including participants with incomplete follow-up information in the analysis. No formal interim analyses are planned.

Sensitivity analyses will be conducted to examine the impact of missing data due to causes other than death, using worst-case and other single imputation on conclusions. Since only participants successfully completing the initial three months falls diary collection period will be entered into the main trial, this should minimise loss of diary information on which the primary outcome is based.

Pre-planned subgroup analyses of the primary outcome will be performed with subgroups of participants defined by disease severity, phenotype and cognitive impairment, with subgroups specified without reference to the treatment allocation variable and before the blind is broken. The significance of any difference in treatment effect between subgroups, will be examined in a test for interaction. We will also examine the effect of the intervention separately in each centre. The extent of intervention that was received by participants will be described. A secondary per-protocol analysis of the main treatment effect will be conducted, restricting the participants included in the intervention group to those receiving an adequate extent of the intervention, to be decided a priori. If we fail to show a statistically significant difference in the primary ITT analysis, the per-protocol analysis will help in understanding whether the lack of significance resulted from dilution of the treatment effect. Exploratory analysis of the outcome in relation to the main components of the intervention will also be undertaken.

### Economic analysis

Readily available unit costs [40] will be attached to all items of resource use and a mean cost per participant estimated. QALYs will be calculated using EQ-5D with incremental cost and incremental benefits (effectiveness and utility) reported within an incremental cost-effectiveness ration (ICER). In addition, in line with the primary outcome measure, the economic evaluation will estimate the incremental cost-per-fall averted. Carer QOL will be calculated and reported separately. The evaluation will adhere to guidelines for good economic evaluation practice [41,42]. Analyses of costs and effects will be undertaken in Stata adhering to good practice for economic evaluations alongside clinical trials [43,44]. Missing cost and QOL data will be explored by employing multiple imputation methods [45,46] using alternative configurations of baseline covariates. The economic evaluation will be reported according to recent guidance [47].

### Nested qualitative study

A longitudinal qualitative study will be conducted alongside the main trial to explore the impact of Parkinson’s on daily life and the expectations and experiences of participants allocated to the intervention study arm. The study will draw on the principles of grounded theory [48,49] and use a maximum variation sampling strategy [50] addressing age (≤ 80 years; > 80 years), gender, falls in previous year (1 fall; 2–10 falls; > 10 falls), disease severity (each of Hoehn and Yahr stages 1–4) and centre (Southampton, Portsmouth, Bournemouth, Exeter). Semi-structured interviews [51] using a topic guide will be conducted with forty participants (ten from each study site) or as many as needed to ensure data saturation, from the intervention arm of the study, in their own home. Each participant will be interviewed twice, firstly after screening but before the intervention commences, and secondly, six months after completion of the intervention. All interviews will be digitally recorded, transcribed verbatim and supplemented by a researcher diary [52]. Analysis will comprise initial, focused, axial and theoretical coding in order to develop interpretive theories conceptualising the experience of participating in PDSAFE. The qualitative researcher will keep a research diary throughout, and will compare coding of initial interviews with a second researcher to promote sensitivity and reflexivity [52]. Supervisory meetings will ensure that preliminary interpretation is open to scrutiny, and that each iteration is shared and tracked. These strategies will help to ensure that rigour is maintained in the conduct and analysis of this sub-study. Along with self-reported dairies of home exercises, the longitudinal qualitative study will contribute more detailed information about factors influencing adherence.

## Discussion

The physical, psychological and economic consequences of falls among PwP are costly in both human and financial terms. This aims of this trial are to establish the effectiveness and cost-effectiveness of a novel, home-delivered physiotherapy intervention (PDSAFE) compared with usual care on risk of falling for people with Parkinson’s who have a history of falling.

PDSAFE is a novel intervention that builds upon the existing literature and targeting known risk factors, being the first trial that uses a novel delivery modus (technology) in conjunction with traditional physiotherapeutic approaches. This will be the largest trial of aiming to reduce falls for PwP to date. The inclusion of a nested qualitative study will aid understanding of the processes and mechanisms of the intervention and outcomes.

If effective, this intervention has the potential to improve the physiotherapy care and therefore patient and service level outcomes for PwP.

## Abbreviations

CES: *Carer Experience Scale*
CRN: *Clinical Research Network*
CSI: *Caregiver Strain Index*
EQ-5D: *EuroQOL -5D*
MMSE: *Mini Mental State Examination*
MoCA: *Montreal Cognitive Assessment*
NIHR: *National Institute of Health Research*
PwP: *People with Parkinson’s*
QALY: *Quality adjusted life years*
QOL: *Quality of life*
